# Conditional Self-Entropy and Conditional Joint Transfer Entropy in Heart Period Variability during Graded Postural Challenge

**DOI:** 10.1371/journal.pone.0132851

**Published:** 2015-07-15

**Authors:** Alberto Porta, Luca Faes, Giandomenico Nollo, Vlasta Bari, Andrea Marchi, Beatrice De Maria, Anielle C. M. Takahashi, Aparecida M. Catai

**Affiliations:** 1 Department of Biomedical Sciences for Health, University of Milan, Milan, Italy; 2 Department of Cardiothoracic, Vascular Anesthesia and Intensive Care, IRCCS Policlinico San Donato, Milan, Italy; 3 BIOtech, Department of Industrial Engineering, University of Trento, Trento, Italy; 4 IRCS PAT-FBK, Trento, Italy; 5 Department of Electronics Information and Bioengineering, Politecnico di Milano, Milan, Italy; 6 Department of Rehabilitation Medicine, IRCCS Fondazione Salvatore Maugeri, Milan, Italy; 7 Department of Physiotherapy, Federal University of São Carlos, São Carlos, São Paulo State, Brazil; Georgia State University, UNITED STATES

## Abstract

Self-entropy (SE) and transfer entropy (TE) are widely utilized in biomedical signal processing to assess the information stored into a system and transferred from a source to a destination respectively. The study proposes a more specific definition of the SE, namely the conditional SE (CSE), and a more flexible definition of the TE based on joint TE (JTE), namely the conditional JTE (CJTE), for the analysis of information dynamics in multivariate time series. In a protocol evoking a gradual sympathetic activation and vagal withdrawal proportional to the magnitude of the orthostatic stimulus, such as the graded head-up tilt, we extracted the beat-to-beat spontaneous variability of heart period (HP), systolic arterial pressure (SAP) and respiratory activity (R) in 19 healthy subjects and we computed SE of HP, CSE of HP given SAP and R, JTE from SAP and R to HP, CJTE from SAP and R to HP given SAP and CJTE from SAP and R to HP given R. CSE of HP given SAP and R was significantly smaller than SE of HP and increased progressively with the amplitude of the stimulus, thus suggesting that dynamics internal to HP and unrelated to SAP and R, possibly linked to sympathetic activation evoked by head-up tilt, might play a role during the orthostatic challenge. While JTE from SAP and R to HP was independent of tilt table angle, CJTE from SAP and R to HP given R and from SAP and R to HP given SAP showed opposite trends with tilt table inclination, thus suggesting that the importance of the cardiac baroreflex increases and the relevance of the cardiopulmonary pathway decreases during head-up tilt. The study demonstrates the high specificity of CSE and the high flexibility of CJTE over real data and proves that they are particularly helpful in disentangling physiological mechanisms and in assessing their different contributions to the overall cardiovascular regulation.

## Introduction

The field of information dynamics is rapidly developing because meaningful quantities useful in multivariate recordings have been devised [1–11] and open access tools for their rapid dissemination have been proposed [12,13]. Among these quantities the most relevant ones are self-entropy (SE) [3,6,7] and transfer entropy (TE) [1] being both factors of the decomposition of the so-called prediction entropy (PE) of an assigned target series in a multivariate dataset [7]. The exploitation of SE and TE is hampered by their rigid definition that is not fully justified given the high flexibility of information-theoretic indexes. Indeed, in the case of SE in a multivariate context the most common definition, i.e. the mutual information between the current value of the dynamics describing the target and its own past values [3], imposes a dependence of SE on the direct dynamical influences of the exogenous sources over the target in addition to the dynamical properties of the destination [3,6,7]. Conversely, the definition of a conditional SE (CSE), eliminating the direct influences of all sources over SE, might provide a more specific index of information internal to the target. In the case of TE, its most common definition in a multivariate context requires the computation of the conditional mutual information between the current value of the target and the past values of a single source given the past of the target and of all other sources [1,11]. With this definition, denoted complete TE or partial TE, the information transfer is quantified from a single source to the destination [1], thus leaving undecided whether it is worth computing the joint TE (JTE) that accounts for the effects of all sources on the destination or a more general measure of the information transfer, such as the conditional JTE (CJTE), that allows one to be flexible in deciding the subset of sources affecting the destination.

The utility of the CSE and CJTE compared to SE and JTE respectively is made clear in the context of the assessment of the cardiovascular control based on spontaneous variations of physiological variables such as heart period (HP), systolic arterial pressure (SAP) and respiration (R). The SE of HP, CSE of HP given SAP and R, JTE from SAP and R to HP, CJTE from SAP and R to HP given SAP and CJTE from SAP and R to HP given R were computed and compared to clarify possibilities and limits of these information-theoretic measures over real data. These quantities are calculated over experimental series obtained in a protocol capable to progressively modifying the dependence of HP dynamics on SAP and R variations as a function of the magnitude of the stimulus [14]. The protocol, i.e. the graded head-up tilt, leading to a sympathetic activation and vagal withdrawal proportional to the inclination of the tilt table [15–17], allows us to monitor the changes of the information-theoretic quantities as a function of the relevance of the challenge and to link their changes to the degree of activation/deactivation of specific physiological mechanisms. The specificity of SE, CSE, JTE and CJTE in describing the underpinning physiological relations is assessed, their meaning is elucidated via the construction of surrogate sets and their physiological correlates are clarified. The information-theoretic quantities are computed under the hypothesis of Gaussanity, stationarity and linear interactions among variables. Even though these hypotheses might appear to be quite restrictive at the first sight, they are largely exploited in multivariate modeling of cardiovascular variability interactions [18–23] and they are usually supposed to hold in the case of well-controlled experimental protocols designed to achieve quasi-stationary conditions, small changes of the variables about the set point (i.e. the mean values of HP, SAP and R) and weak interactions among physiological subsystems.

## Methods

### Open Loop Autoregressive Model with *q* Exogenous Inputs and its Simplified Model structures

Given an effect time series *y* = {*y*(*n*), *n* = 1, …,*N*}, and *q* exogenous driving time series, *x*_*1*_ = {*x*_*1*_(*n*), *n* = 1, …,*N*},…, *x*_*q*_ = {*x*_*q*_(*n*), *n* = 1, …,*N*} where *N* is the series length and *n* is the progressive counter, all series are first normalized to have zero mean and unit variance by subtracting the mean to each sample and by dividing the result by the standard deviation. The set *Ω* = {*y*,*x*_*1*_,…,*x*_*q*_}, formed by the reunion of the subset exclusively including the series *y* (i.e. {*y*}) and the subset collecting all exogenous series *Ω*\*y* = {*x*_*1*_,…,*x*_*q*_}, constitutes the universe of knowledge about the system under study. The series *y* is the assigned effect and describes the behavior of the subsystem defined as destination, while *Ω*\*y* is the set collecting all presumed causes describing the behavior of the subsystems defined as sources and supposed to affect the destination. The open loop autoregressive (AR) model with *q* exogenous (X) inputs (ARX_1_^…^X_*q*_) is defined as

$$y\left(n\right)=A\left(z^{-1}\right)\cdot y\left(n\right)+\sum_{j=1}^{q}B_{j}\left(z^{-1}\right)\cdot x_{j}\left(n\right)+w_{{\text{ARX}}_{1}\cdots {\text{X}}_{q}}\left(n\right)$$ (1)

where $w_{{\text{ARX}}_{1}\cdots {\text{X}}_{q}}$ is a zero mean white Gaussian noise with variance $\lambda_{{\text{ARX}}_{1}\cdots {\text{X}}_{q}}^{2}$, *A*(*z* ^−1^) and *B*_*j*_(*z* ^−1^), with 1≤*j*≤*q*, are polynomials in *z* ^-1^ with constant coefficients and *z* ^-1^ is the unit backward shift operator (i.e. *z* ^-1.^ *y*(*n*) = *y*(*n*-1)) in the *Z*-domain. The polynomial

$$A\left(z^{-1}\right)=\sum_{i=1}^{p}a_{i}\cdot z^{-i}$$ (2)

describes the self-dependence of *y* on *p* past samples of the same series where the *a*_*i*_’s, with 1≤*i*≤*p*, are the constant coefficients of the auto-regression of *y* and the polynomial

$$B_{j}\left(z^{-1}\right)=\sum_{i=0}^{p}b_{ji}\cdot z^{-(i+\tau_{j})}$$ (3)

describes the cross-dependence of *y* on *p*+1 present and past samples of the series *x*_*j*_ where the *b*_*ji*_‘s, with 0≤*i*≤*p*, are the constant coefficients of the regression of *y* on *x*_*j*_ and *τ*_*j*_ is the delay from *x*_*j*_ to *y*.

Three structures, all derived from the ARX_1_^…^X_*q*_ model, are of interest in this study: i) the ARX_1_^…^X_*j*-*r*_X_*j*+*s*_^…^X_*q*_ model, with *s* and *r* integers with 1≤*r*≤*j* and 1≤*s*≤*q*-*j*+1, that disregards a block of exogenous causes (i.e. *x*_*j*-*r*+1_,…,*x*_*j*+*s*-1_) by ignoring the regression of *y* on *x*_*j*-*r*+1_,…,*x*_*j*+*s*-1_; ii) the AR model that disregards all regressions of *y* on *x*_*j*_ with 1≤*j*≤*q*; iii) the X_1_^…^X_*q*_ model that disregards only the AR part by ignoring the auto-regression of *y*.

### Prediction of *y* Based on the ARX_1_…X_q_ Model and its Simplified Model Structures

The one-step-ahead prediction of *y*, $\hat{y}\left(n/n-1\right)$, based on the ARX_1_^…^X_*q*_ model is given by

$$\hat{y}\left(n/n-1\right)=\hat{A}\left(z^{-1}\right)\cdot \text{y}\left(n\right)+\sum_{j=1}^{q}{\hat{B}}_{j}\left(z^{-1}\right)\cdot x_{j}\left(n\right)$$ (4)

where the coefficients of $\hat{A}\left(z^{-1}\right)$ and ${\hat{B}}_{j}\left(z^{-1}\right)$ are estimated according to an optimization criterion (here the least squares approach minimizing the variance of $w_{{\text{ARX}}_{1}\cdots {\text{X}}_{q}}$) [24]. Basically $\hat{y}\left(n/n-1\right)$ is deterministically obtained by filtering *y* and *x*_*j*_ with 1≤*j*≤*q* with $\hat{A}\left(z^{-1}\right)$ and ${\hat{B}}_{j}\left(z^{-1}\right)$ respectively. The one-step ahead prediction of *y* based on the considered simplified versions of the ARX_1_^…^X_*q*_ model (i.e. the ARX_1_^…^X_*j*-*r*_X_*j*+*s*_^…^X_*q*_ with *r*,*s*≥1, AR and X_1_^…^X_*q*_ structures) can be analogously obtained after a new identification of the polynomials via minimization of the variance of their white noises (i.e. $w_{{\text{ARX}}_{1}\cdots {\text{X}}_{j-r}{\text{X}}_{j+s}\cdots {\text{X}}_{q}}$, *w*_AR_ and $w_{{\text{X}}_{1}\cdots {\text{X}}_{q}}$ respectively). Defined the prediction error as the difference between *y*(*n*) and its one-step-ahead prediction, $\hat{y}\left(n/n-1\right)$, the inability of the model to describe the dynamics of *y* is quantified by the variance of the prediction error. It is bounded between 0 and the variance of *y*, σ^2^. Given the normalization of the series as reported in the previous Section, the variance of the prediction error actually ranges between 0 and 1, where 0 indicates perfect prediction (the entire σ^2^ is explained by the model) and 1 indicates null prediction (no fraction of σ^2^ is explained by the model). In the following we will indicate as $\sigma_{{\text{ARX}}_{1}\cdots {\text{X}}_{q}}^{2}$, $\sigma_{{\text{ARX}}_{1}\cdots {\text{X}}_{j-r}{\text{X}}_{j+s}\cdots {\text{X}}_{q}}^{2}$, $\sigma_{\text{AR}}^{2}$ and $\sigma_{{\text{X}}_{1}\cdots {\text{X}}_{q}}^{2}$ the variance of the prediction error of the ARX_1_^…^X_*q*_, ARX_1_^…^X_*j*-*r*_X_*j*+*s*_^…^X_*q*_, X_1_^…^X_*q*_ and AR model structures respectively.

### Linear Gaussian Approximation of the Information-Theoretic Quantities

**Fig 1:**
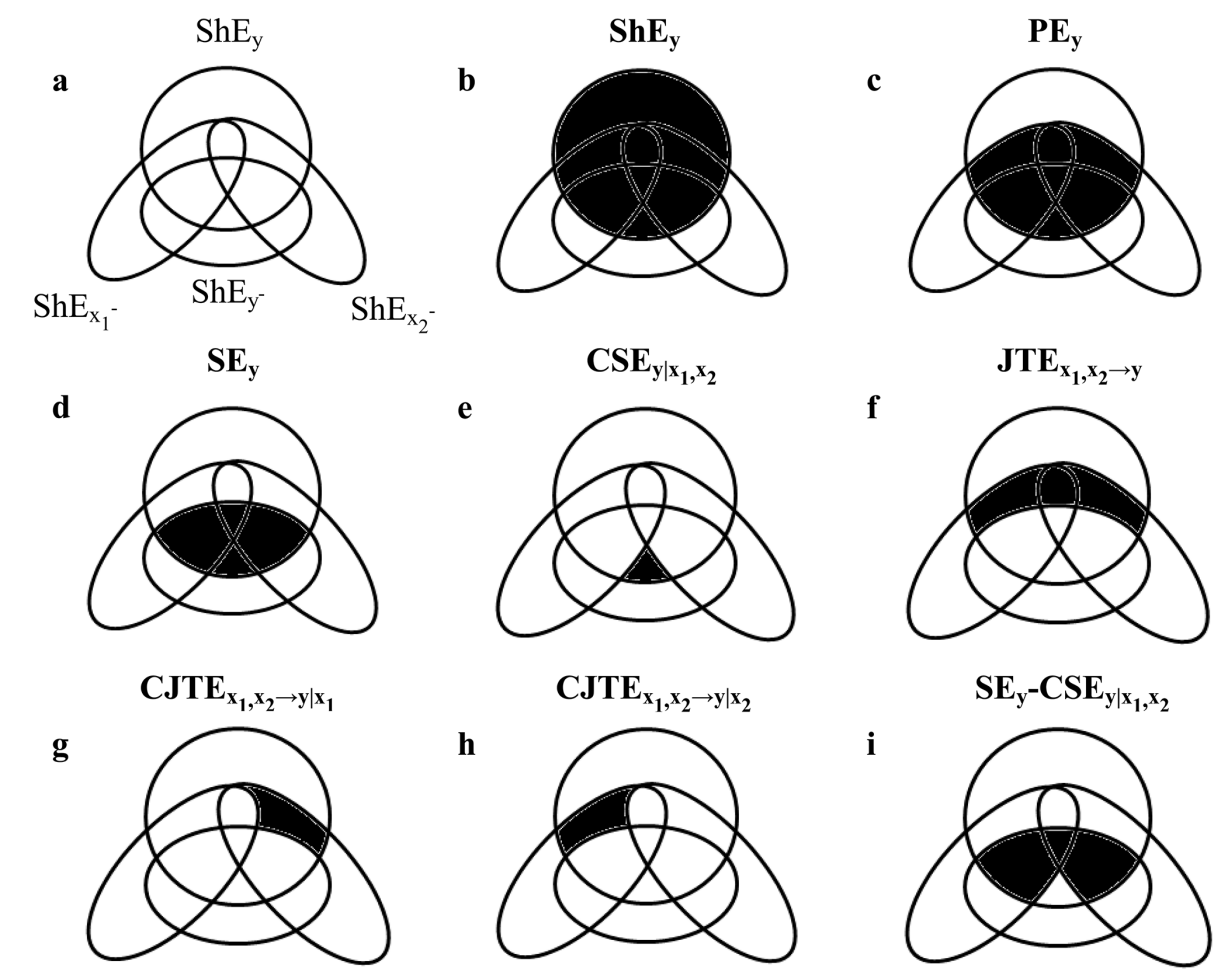
Graphical Representation of the Information-Theoretic Quantities in *Ω* = {*y*,*x*_1_,x_2_}. A mnemonic Venn diagram of the prominent information-theoretic quantities that are computed in the context of this study. The diagram is devised to represent in the information domain the dependency of the uncertainty associated to the current value of *y* = {*y(n*), *n* = 1002C…, *N*} quantified by the Shannon entropy (ShE) of *y* (ShE_**y**_) on the information associated to past values of the assigned effect series *y* ^*-*^ = {[*y(n*-1), …, *y(n*-*p*)], *n* = *p*+1, …,*N*} quantified by ${\text{ShE}}_{y^{-}}$, and of two presumed causes *x*_***1***_^*-*^ = {[*x*_***1***_ *(n*-*τ*_***1***_),…, *x*_***1***_ *(n*-*τ*_***1***_-*p*)], *n* = *τ*_***1***_+*p*+1, …,*N*} and *x*_***2***_^*-*^ = {[*x*_***2***_ *(n*-*τ*_***2***_),…, *x*_***2***_ *(n*-*τ*_***2***_-*p*)], *n* = *τ*_***2***_+*p*+1, …,*N*} quantified by ${\text{ShE}}_{{\text{x}}_{1}^{-}}$ and ${\text{ShE}}_{{\text{x}}_{2}^{-}}$ respectively. The four intersecting circles (or ellipses) represent ShE_*y*_, ${\text{ShE}}_{y^{-}}$, ${\text{ShE}}_{{\text{x}}_{1}^{-}}$ and ${\text{ShE}}_{{\text{x}}_{2}^{-}}$ (a). Notable quantities for this contribution are highlighted by filling in black the relevant areas at the interception of the circles (or ellipses): ShE_*y*_ (b), PE_*y*_ (c), SE_*y*_ (d), ${\text{CSE}}_{y|x_{1},x_{2}}$ (e), ${\text{JTE}}_{x_{1},x_{2}\to y}$ (f), ${\text{CJTE}}_{x_{1},x_{2}\to y|x_{1}}$ (g), ${\text{CJTE}}_{x_{1},x_{2}\to y|x_{2}}$ (h), and SE_*y*_-${\text{CSE}}_{y|x_{1},x_{2}}$ (i).

Under the hypothesis of joint Gaussian distribution of the variables (i.e. any subset of consecutive samples derived from *Ω* has a joint Gaussian distribution), the SE, CSE, JTE and CJTE can be exactly computed from the multivariate linear regression representation of the dynamics of *y* given by the ARX_1_^…^X_*q*_ model [4,7,25,26] as listed below. The full list of the information-theoretic quantities computed in the context of this study is given in Table 1 and their schematic representation is given for *q* = 2 in Fig 1.

**Table 1:** Synopsis of the Information-Theoretic Quantities Considered in the Study.

Acronym	Definition
ShE_*y*_	Overall amount of uncertainty about the present of *y* [27]
PE_*y*_	Amount of uncertainty about the present of *y* explained by past values of all signals in *Ω* [7,10]
SE_*y*_	Amount of uncertainty about the present of *y* explained by past values of y [3,6]
${\text{CSE}}_{y|x_{1},\cdots ,x_{q}}$	SE_*y*_ conditioned on all exogenous signals
${\text{JTE}}_{x_{1},\cdots ,x_{q}\to y}$	Amount of uncertainty about the present of *y* explained by past values of all exogenous signals above and beyond that resolved by past values of *y*
${\text{CJTE}}_{x_{1},\cdots ,x_{q}\to y|x_{1},\cdots ,x_{j\text{-}r},x_{j+s},\cdots ,x_{q}}$	Portion of the ${\text{JTE}}_{x_{1},\cdots ,x_{q}\to y}$ solely attributable to the block of exogenous signals {*x*_*j*-*r*+1_,…,*x*_*j*+*s*-1_}
${\text{CJTE}}_{x_{1},\cdots ,x_{q}\to y|x_{1},\cdots ,x_{j\text{-}1},x_{j+1},\cdots ,x_{q}}$	Portion of the ${\text{JTE}}_{x_{1},\cdots ,x_{q}\to y}$ solely attributable to past values of *x*_*j*_ and coincident with ${\text{TE}}_{x_{j}\to y}$ in *Ω* [1,11,28]

ShE = Shannon entropy; PE = prediction entropy; SE = self-entropy; CSE = Conditional SE; TE = transfer entropy; JTE = joint TE; CJTE = conditional JTE.

We define as prediction entropy (PE) of *y*

$${\text{PE}}_{y}=\frac{1}{2}\text{log}\frac{\sigma^{2}}{\sigma_{{\text{ARX}}_{1}\cdots {\text{X}}_{q}}^{2}},$$ (5)

where log is the natural logarithm, the ratio of the variance of *y* on that of the prediction error of the ARX_1_^…^X_*q*_ model. PE measures the reduction of the uncertainty about *y*, quantified by Shannon entropy of *y* (ShE_*y*_) (Fig 1B) owing to the ARX_1_^…^X_*q*_ fitting (Fig 1C). PE_*y*_ is bounded between 0 and ShE_*y*_ being 0.5^.^log(2πeσ^2^) under the hypothesis of Gaussian distribution [27].

SE of *y*, SE_*y*_, can be calculated as the ratio of the variance of y on that of the prediction error of the AR model as

$${\text{SE}}_{y}=\frac{1}{2}\text{log}\frac{\sigma^{2}}{\sigma_{\text{AR}}^{2}}$$ (6)

representing the reduction of the uncertainty about *y* owing to the AR fitting (Fig 1D). SE_*y*_ is bounded between 0 and PE_*y*_.

CSE of *y* conditioned on all assigned causes, ${\text{CSE}}_{y|x_{1},\cdots ,x_{q}}$, is calculated as the ratio of the prediction error variance of the X_1_^…^X_*q*_ model on that of the ARX_1_^…^X_*q*_ model as

$${\text{CSE}}_{y|x_{1},\cdots ,x_{q}}=\frac{1}{2}\text{log}\frac{\sigma_{{\text{X}}_{1}\cdots {\text{X}}_{q}}^{2}}{\sigma_{{\text{ARX}}_{1}\cdots {\text{X}}_{q}}^{2}}$$ (7)

representing the reduction of the uncertainty about *y* when *y* is described according to the X_1_^…^X_*q*_ model owing to the consideration of past values of *y* in addition to those of all presumed causes X_1_^…^X_*q*_ (Fig 1E).

Joint TE (JTE) from all the presumed causes to *y*, ${\text{JTE}}_{x_{1},\cdots ,x_{q}\to y}$, is calculated as the ratio of the prediction error variance of the AR model on that of the ARX_1_^…^X_*q*_ model as

$${\text{JTE}}_{x_{1},\cdots ,x_{q}\to \text{y}}=\frac{1}{2}\text{log}\frac{\sigma_{\text{AR}}^{2}}{\sigma_{{\text{ARX}}_{1}\cdots {\text{X}}_{q}}^{2}}$$ (8)

representing the reduction of the uncertainty about *y* when *y* is described according to the AR model owing to the consideration of all presumed causes X_1_^…^X_*q*_ in addition to the past values of *y* (Fig 1F). ${\text{JTE}}_{x_{1},\cdots ,x_{q}\to y}$ is bounded between 0 and PE_*y*_. The ${\text{JTE}}_{x_{1},\cdots ,x_{q}\to y}$ can be rendered more specific by conditioning ${\text{JTE}}_{x_{1},\cdots ,x_{q}\to y}$ to a subset of causes chosen in *Ω*\*y*. For example, the conditional ${\text{JTE}}_{x_{1},\cdots ,x_{q}\to y}$ given *x*_1_,…,*x*_*j*-*r*_,…,*x*_*j*+*s*_,…,*x*_*q*_ with 1≤*r*≤*j* and 1≤*s*≤*q*-*j*+1

$${\text{CJTE}}_{x_{1},\cdots ,x_{q}\to y|x_{1},\cdots ,x_{j-r},x_{j+s},\cdots ,x_{q}}=\frac{1}{2}\text{log}\frac{\sigma_{{\text{ARX}}_{1}\cdots {\text{X}}_{j-r}{\text{X}}_{j+s}\cdots {\text{X}}_{q}}^{2}}{\sigma_{{\text{ARX}}_{1}\cdots {\text{X}}_{q}}^{2}},$$ (9)

computed as the ratio of the prediction error variance of the ARX_1_^…^X_*j*-*r*_X_*j*+*s*_^…^X_*q*_ model on that of the ARX_1_^…^X_*q*_ model, represents the reduction of the uncertainty about *y* when *y* is described according to the ARX_1_^…^X_*j*-*r*_X_*j*+*s*_^…^X_*q*_ model owing to the consideration of past values of the excluded set of causes *Ω*\*y*,*x*_1_,…,*x*_*j*-*r*_,…,*x*_*j*+*s*_,…,*x*_*q*_ = {*x*_*j*-*r*+1_,…,*x*_*j*+*s*-1_} in addition to past values of *y* and the presumed causes *x*_1_,…,*x*_*j*-*r*_,…,*x*_*j*+*s*_,…,*x*_*q*_. When the ${\text{CJTE}}_{x_{1},\cdots ,x_{q}\to y|x_{1},\cdots ,x_{j-r},x_{j+s},\cdots ,x_{q}}$ is computed with *r* = 1 and *s* = 1, it becomes

$${\text{CJTE}}_{x_{1},\cdots ,x_{q}\to y|x_{1},\cdots ,x_{j-1},x_{j+1},\cdots ,x_{q}}=\frac{1}{2}\text{log}\frac{\sigma_{{\text{ARX}}_{1}\cdots {\text{X}}_{j-1}{\text{X}}_{j+1}\cdots {\text{X}}_{q}}^{2}}{\sigma_{{\text{ARX}}_{1}\cdots {\text{X}}_{q}}^{2}}$$ (10)

representing the reduction of the uncertainty about *y* when *y* is described according to the ARX_1_^…^X_*j*-1_X_*j*+1_^…^X_*q*_ model owing to the consideration of past values of the excluded cause *x*_*j*_ in addition to past values of *y* and the presumed causes *x*_*1*_,…,*x*_*j*-1_,*x*_*j*+1_,…,*x*_*q*_ (Fig 1G and 1H). This quantity is commonly indicated as complete TE [1,28] or partial TE [11] and denoted as ${\text{TE}}_{x_{j}\to y}$ in *Ω* = {*y*,*x*_*1*_,…,*x*_*q*_}. Here we prefer the acronym ${\text{CJTE}}_{x_{1},\cdots ,x_{q}\to y|x_{1},\cdots ,x_{j-1},x_{j+1},\cdots ,x_{q}}$ to stress, even formally, the relation with ${\text{JTE}}_{x_{1},\cdots ,x_{q}\to y}$ (i.e. ${\text{JTE}}_{x_{1},\cdots ,x_{q}\to y}$ provides the upper bound of any CJTE in *Ω* being the lower bound 0) and the higher generality of CJTE compared to complete ${\text{TE}}_{x_{j}\to y}$ given the more general partialling condition subsuming the most widely utilized one in ${\text{TE}}_{x_{j}\to y}$.

## Experimental Protocol and Data Analysis

### Ethics Statement

The study was performed according to the Declaration of Helsinki and it was approved by the Human Research Ethics Committee of the “L. Sacco” Hospital and Department of Biomedical and Clinical Sciences, University of Milan, Milan, Italy [14]. A written informed consent was obtained from all subjects.

### Experimental Protocol

We exploited a set of recordings collected during an experimental protocol planned to study the effect of a graded postural challenge on the cardiac baroreflex control [14]. Briefly, we studied 19 nonsmoking healthy humans aged from 21 to 48 years (median age = 30 years, 8 males). All subjects had neither history nor clinical evidence of any disease. They did not take any medication. They refrained from consuming any caffeine or alcohol-containing beverages in the 24h before the recording. All experiments were performed in the morning. The subjects were on the tilt table supported by two belts at the level of thigh and waist respectively and with both the feet touching the footrest of the tilt table. During the entire protocol the subjects breathed spontaneously but they were not allowed to talk.

ECG (lead II), continuous plethysmographic arterial pressure (Finometer MIDI, Finapres Medical Systems, The Netherlands) and respiratory movements via a thoracic belt (Marazza, Monza, Italy) were recorded. Signals were sampled at 300 Hz. The arterial pressure was measured from the middle finger of the left hand being maintained at the level of heart by fixing the subject’s arm to his/her thorax during the upright position. All experimental sessions of the protocol included three periods in the same order: 1) 7 minutes at REST; 2) 10 minutes during passive head-up tilt (T); 3) 8 minutes of recovery. The inclination of the tilt table, expressed as degrees, was randomly chosen within the set {15,30,45,60,75,90} (T15, T30, T45, T60, T75, T90). Each subject completed the sequence of tilt table angles without experiencing any sign of pre-syncope. The arterial pressure signal was cross-calibrated in each session using a measure provided by a sphygmomanometer at the onset of REST. The auto-calibration procedure of the arterial pressure device was switched off after the first automatic calibration at the onset of the session. Analyses were performed after about 2 minutes from the start of each period.

### Extraction of the Beat-to-Beat Variability Series

After detecting all R-waves on the ECG and locating their peak using parabolic interpolation, HP was approximated as the temporal distance between two consecutive parabolic apexes. The maximum of arterial pressure inside of the *n*-th HP, HP(*n*), was taken as the *n*-th SAP, SAP(*n*). The signal of the thoracic movements was down-sampled once per cardiac beat at the occurrence of the first R-wave peak delimiting HP(*n*), thus obtaining the *n*-th R measure, R(*n*). HP(*n*), SAP(*n*) and R(*n*) were expressed in ms, mmHg and arbitrary units (a.u.) respectively. The automatic detections of the R-waves and SAP peaks were visually checked by a trained physician. After extracting the series HP = {HP(*n*), *n* = 1,…,*N*}, SAP = {SAP(*n*), *n* = 1,…,*N*} and R = {R(*n*), *n* = 1,…,*N*}, where *n* is the progressive cardiac beat counter and *N* is the total cardiac beat number, 256 consecutive, synchronous, HP, SAP and R measures were chosen inside the REST and T periods, thus focusing short-term cardiovascular regulatory mechanisms [29]. The random selection of the onset of analysis within the overall REST and T periods made this preprocessing step operator-independent. The full set of original HP, SAP and R series is available from the Dryad URL http://dx.doi.org/10.5061/dryad.b32v1.

### Construction of Surrogates

We tested the null hypothesis of HP-SAP and HP-R coupling. This test was performed by creating three sets of surrogates.

The first set was composed by the original SAP and R series, while the HP sequence was substituted with a series obtained by randomly shuffling the HP samples [30]. The shuffling procedure was performed according to one of the N! permutations of the HP samples. As a consequence these surrogates preserved the distribution of the original series, SAP and R series were fully uncoupled to HP dynamics and solely SAP and R series maintained the original repetitive temporal structures. This surrogate will be referred to as HP shuffled surrogate in the following.

The second set was composed by the original HP series, while SAP and R sequences were substituted with isospectral isodistributed surrogates obtained by preserving distributions and power spectra of the original SAP and R series respectively, while phases were modified by adding uniformly distributed random numbers ranging from 0 to 2π [31]. An iteratively-refined amplitude-adjusted Fourier transform-based procedure was exploited [32]. As a consequence SAP and R surrogates preserved exactly the distribution of the original series, while the power spectrum was the best approximation of the original power spectrum according to the number of iterates (here 100). Phase randomization procedure assured the uncoupling of SAP and R to the original HP series [30,33]. This surrogate will be referred to as SAP-R isospectral surrogate in the following.

The third set was composed by time-shifted versions of the original series [34]. While the HP series was left unmodified, the SAP and R sequences were shifted according to a delay much larger than the maximal order of the multivariate model (i.e. 50 cardiac beats), thus destroying the short-term temporal correspondence of the SAP and R samples to HP values. The values at the end of the SAP and R sequences were wrapped to their onset. This surrogate will be referred to as time-shifted surrogate in the following.

### Calculation of the Information-Theoretic Indexes

The HP series was modeled via ARX_1_X_2_, ARX_1_, ARX_2_, X_1_X_2_, and AR models where X_1_ = SAP and X_2_ = R. The delays from SAP and R to HP, τ_SAP_ and τ_R_, were set to 0 to allow the description of the fast vagal reflex (within the mean HP) capable to modify HP in response to changes of SAP and R [18,35,36]. The coefficients were identified via traditional least squares approach and Cholesky decomposition method [24,37]. The AR and X parts of the model have the same model order *q* that was optimized in the range from 4 to 16 according to the Akaike figure of merit for multivariate processes [38] over the most complex model structure (i.e. the ARX_1_X_2_, model). The whiteness of the HP prediction error and its mutual uncorrelation, even at zero lag, with SAP and R series was checked over the same model [37,39]. All remaining model structures were separately identified from the data using the optimal model order estimated for the ARX_1_X_2_ model. After the identification of the model coefficients the variances of the prediction errors $\sigma_{\text{AR}}^{2}$, $\sigma_{{\text{X}}_{1}{\text{X}}_{2}}^{2}$, $\sigma_{{\text{ARX}}_{1}}^{2}$, $\sigma_{{\text{ARX}}_{2}}^{2}$ and $\sigma_{{\text{ARX}}_{1}{\text{X}}_{2}}^{2}$ were computed and the indexes PE_HP_, SE_HP_, CSE_HP|SAP,R_, JTE_SAP,R→HP_, CJTE_SAP,R→HP|SAP_ and CJTE_SAP,R→HP|R_ were evaluated according to the formulas reported in the Section *Linear Gaussian approximation of the information-theoretic quantities*. Indexes were computed over the original series and surrogates. If the values derived from the original data were significantly different from those obtained from the surrogate sets the null hypothesis of HP-SAP and HP-R coupling was rejected.

### Statistical Analysis

We performed one-way repeated measures analysis of variance (Dunn’s test for multiple comparisons) to test the difference of PE_HP_ between original series and surrogates. Two-way repeated measures analysis of variance (Holm-Sidak test for multiple comparisons) was utilized to test the difference between indexes within original series and between original series and surrogates within the same type of index. Linear regression analysis of PE_HP_, SE_HP_, CSE_HP|SAP,R_, JTE_SAP,R→HP_, CJTE_SAP,R→HP|SAP_ and CJTE_SAP,R→HP|R_ on tilt table angles was carried out over original data and surrogates. Pearson product moment correlation was calculated. Statistical analysis was carried out using a commercial statistical program (Sigmastat, ver.3.0.1, Systat Software, San Jose, California). A p<0.05 was always considered as significant.

## Results

**Fig 2:**
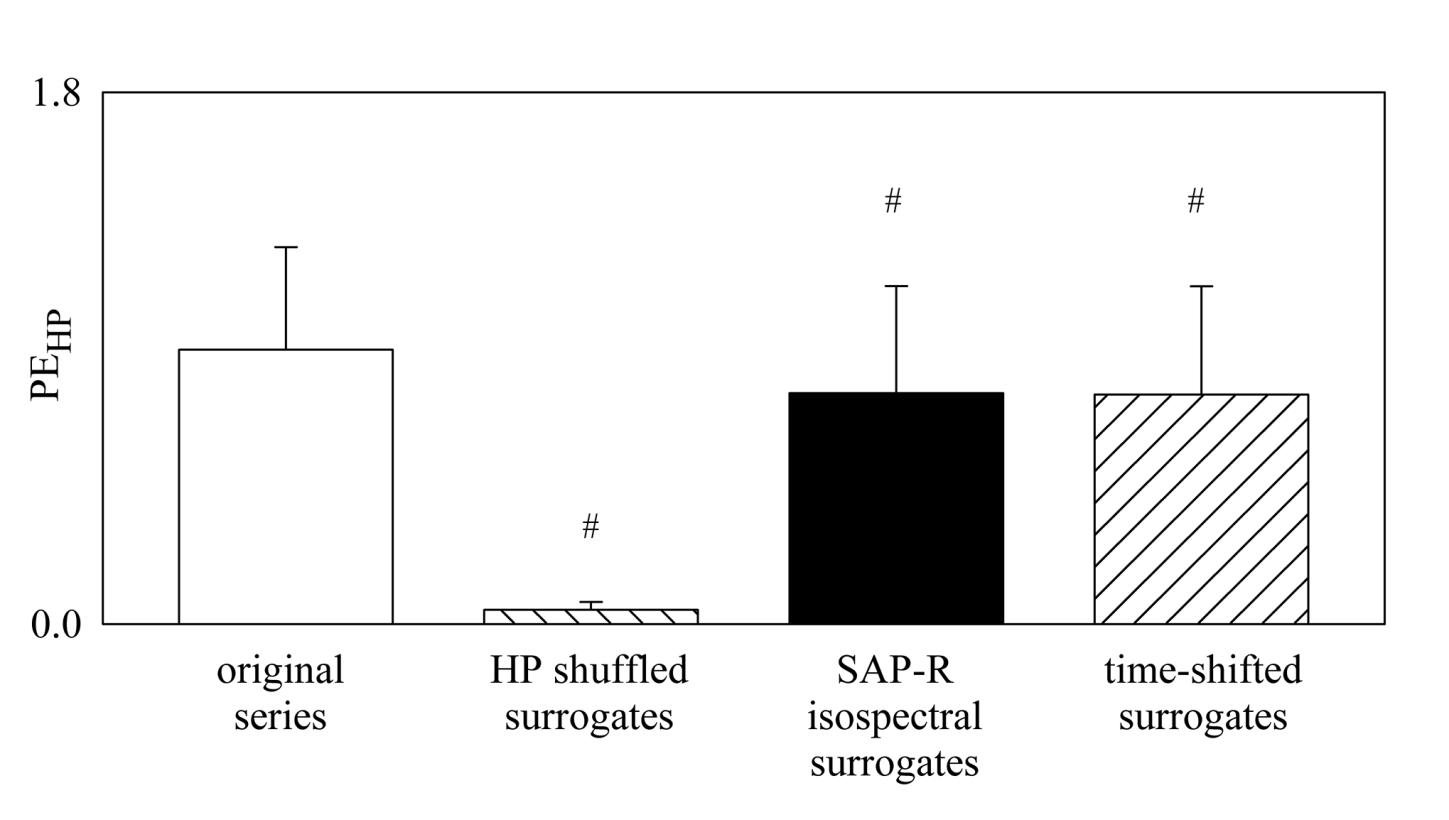
PE_HP_ Derived from Original Data and Surrogates. Bar graph compares PE_**HP**_ computed during graded head-up tilt protocol over the original data (white bar), HP shuffled surrogates (backslash pattern bar), SAP-R isospectral surrogates (black bar) and time-shifted surrogates (slash pattern bar). The values are pooled together independently of the tilt table inclination and reported as mean plus standard deviation. The symbol # indicates a significant difference between original and surrogate series.

The bar graph shown in Fig 2 compares PE_HP_ computed over the original data (white bar), HP shuffled surrogates (backslash pattern bar), SAP-R isospectral surrogates (black bar) and time-shifted surrogates (slash pattern bar). The values were pooled together independently of tilt table inclination. PE_HP_ computed over the original data was significantly larger than that computed over surrogates.

**Fig 3:**
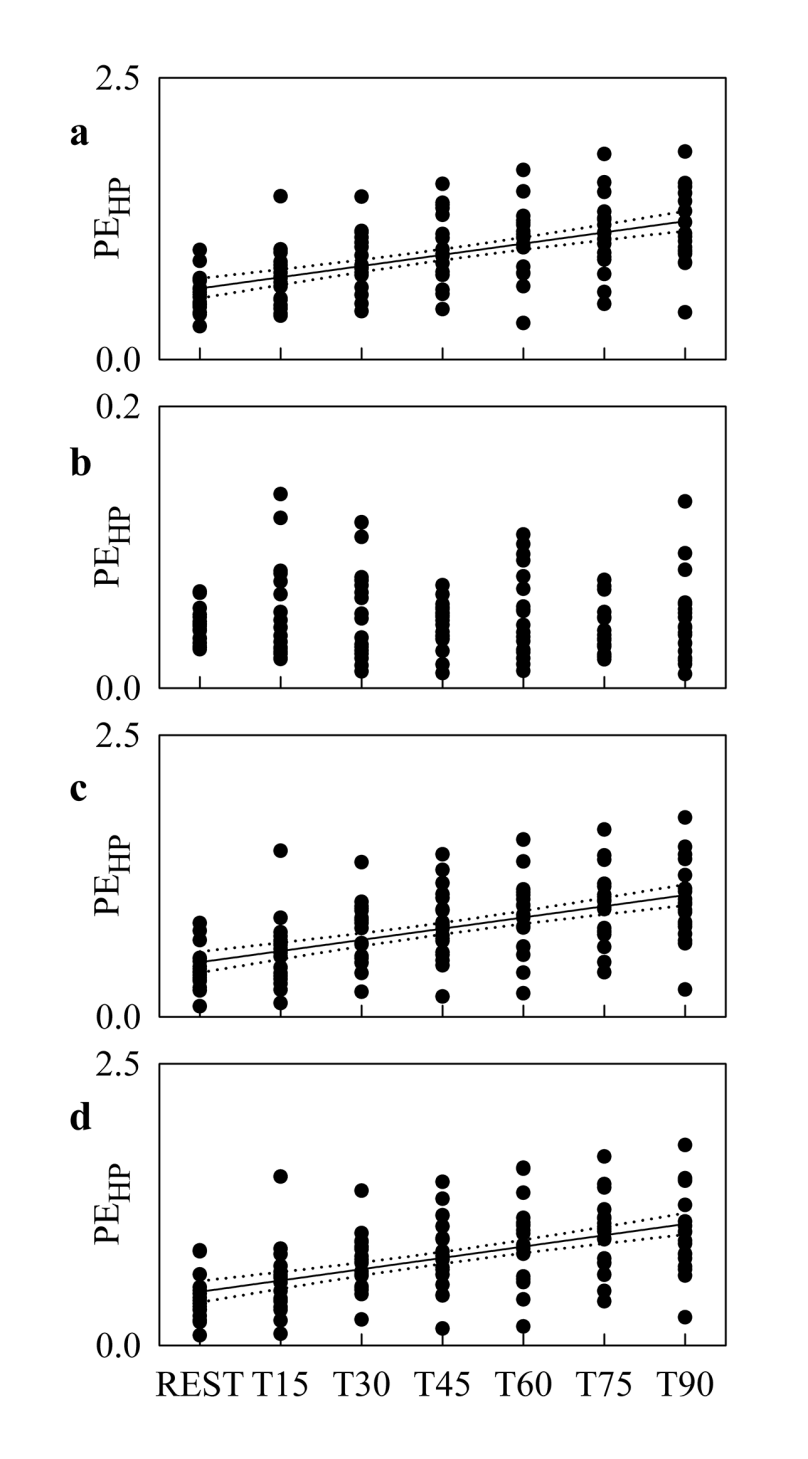
PE_HP_ during Graded Head-up Tilt. Individual values (solid circles) of PE_**HP**_ as a function of the tilt table inclination computed over the original data (a), HP shuffled surrogates (b), SAP-R isospectral surrogates (c) and time-shifted surrogates (d). When the slope of the regression line is significantly larger then 0, the linear regression (solid line) and its 95 percent confidence interval (dotted lines) are plotted as well. A significant positive correlation on tilt table angles is found over the original data (a), SAP-R isospectral surrogates (c) and time-shifted surrogates (d).

**Fig 4:**
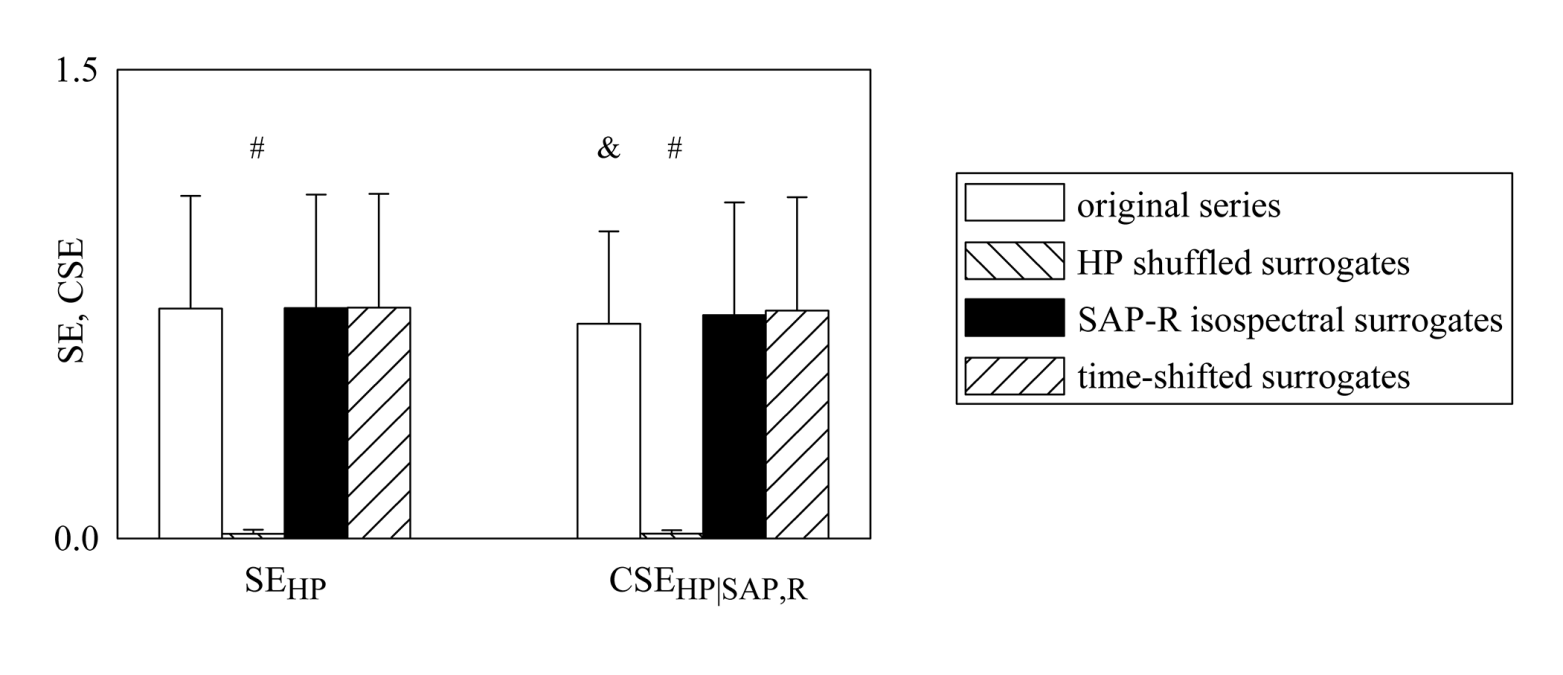
Comparison between SE_HP_ and CSE_HP|SAP,R_. Grouped bar graph compares SE_**HP**_ and CSE_**HP|SAP,R**_ computed during graded head-up tilt protocol over the original data (white bars), HP shuffled surrogates (backslash pattern bars), SAP-R isospectral surrogates (black bars), and time-shifted surrogates (slash pattern bars). The values are pooled together independently of the tilt table inclination and reported as mean plus standard deviation. Within the same index (i.e. SE_**HP**_ or CSE_**HP|SAP,R**_) the symbol # indicate a significant difference between original and surrogate series. Within the original data the symbol & indicates a significant difference between SE_**HP**_ and CSE_**HP|SAP,R**_.

Fig 3 shows the individual values (solid circles) of PE_HP_ as a function of the tilt table inclination in the original data (Fig 3A), HP shuffled surrogates (Fig 3B), SAP-R isospectral surrogates (Fig 3C) and time-shifted surrogates (Fig 3D). The linear regression (solid line) and its 95 percent confidence interval (dotted lines) are plotted when the slope of the regression line is significantly larger than 0. When the linear regression analysis was performed over the original data (Fig 3A), SAP-R isospectral surrogates (Fig 3C) and time-shifted surrogates (Fig 4D), a significant positive correlation of PE_HP_ on tilt table angles was found (r = 0.575, p = 4.548^.^10^−13^, r = 0.552, p = 5.742^.^10^−12^ and r = 0.55, p = 7.07^.^10^−12^ respectively). Conversely, no linear relation of PE_HP_ on the magnitude of the orthostatic challenge was detected in the case of HP shuffled surrogates (Fig 3B).

The grouped bar graph shown in Fig 4 compares SE_HP_ and CSE_HP|SAP,R_ computed during head-up tilt protocol over the original data (white bars), HP shuffled surrogates (backslash pattern bars), SAP-R isospectral surrogates (black bars) and time-shifted surrogates (slash pattern bars). The values were pooled together independently of tilt table inclination. Both SE_HP_ and CSE_HP|SAP,R_ were significantly different from 0 in both original data, SAP-R isospectral and time-shifted surrogates, while they were negligible in the case of HP shuffled surrogates. Within the original data we found that SE_HP_ was larger than CSE_HP|SAP,R_. Within the same index (i.e. SE_HP_ or CSE_HP|SAP,R_) both markers computed over the original data were significantly different from those derived from HP shuffled surrogates but similar to those derived from the other surrogate types.

**Fig 5:**
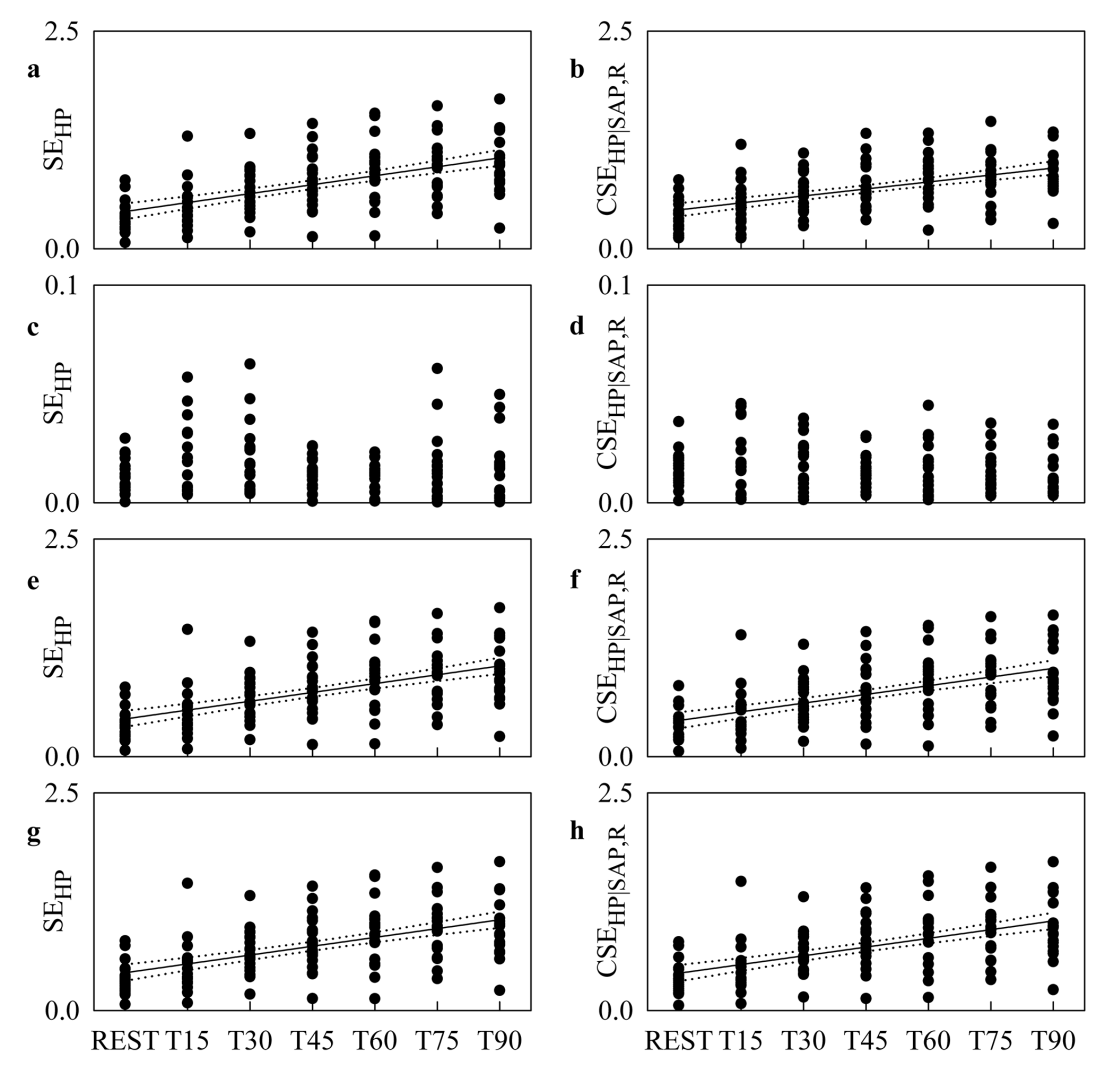
SE_HP_ and CSE_HP|SAP,R_ during Graded Head-up Tilt. Individual values (solid circles) of SE_**HP**_ and CSE_**HP|SAP,R**_ as a function of the tilt table inclination computed over the original data (a,b), HP shuffled surrogates (c,d), SAP-R isospectral surrogates (e,f) and time-shifted surrogates (g,h). When the slope of the regression line is significantly larger then 0, the linear regression (solid line) and its 95 percent confidence interval (dotted lines) are plotted as well. A significant positive correlation on tilt table angles is found over the original data (a,b), SAP-R isospectral surrogates (e,f) and time-shifted surrogates (g,h) in the case of both SE_**HP**_ and CSE_**HP|SAP,R**_.

Fig 5 shows the individual values (solid circles) of SE_HP_ and CSE_HP|SAP,R_ as a function of the tilt table inclination in the original data (Fig 5A and 5B), HP shuffled surrogates (Fig 5C and 5D), SAP-R isospectral surrogates (Fig 5E and 5F) and time-shifted surrogates (Fig 5G and 5H). The linear regression (solid line) and its 95 percent confidence interval (dotted lines) are plotted when the slope of the regression line is significantly larger than 0. When the linear regression analysis was performed over the original data, SE_HP_ (Fig 5A) and CSE_HP|SAP,R_ (Fig 5B) are significantly and positively correlated with tilt table angles (r = 0.572, p = 6.135^.^10^−13^ and r = 0.544, p = 1.336^.^10^−11^). The same finding held when the linear regression analysis was performed over the SAP-R isospectral surrogates (Fig 5E: r = 0.56, p = 2.313^.^10^−12^ and Fig 5F: r = 0.555, p = 4.034^.^10^−12^) and over the time-shifted surrogates (Fig 5G: r = 0.559, p = 2.808^.^10^−12^ and Fig 5H: r = 0.554, p = 4.7^.^10^−12^). Conversely, no linear relation of SE_HP_ and CSE_HP|SAP,R_ on the magnitude of the orthostatic challenge was detected in the case of HP shuffled surrogates (Fig 5C and 5D).

**Fig 6:**
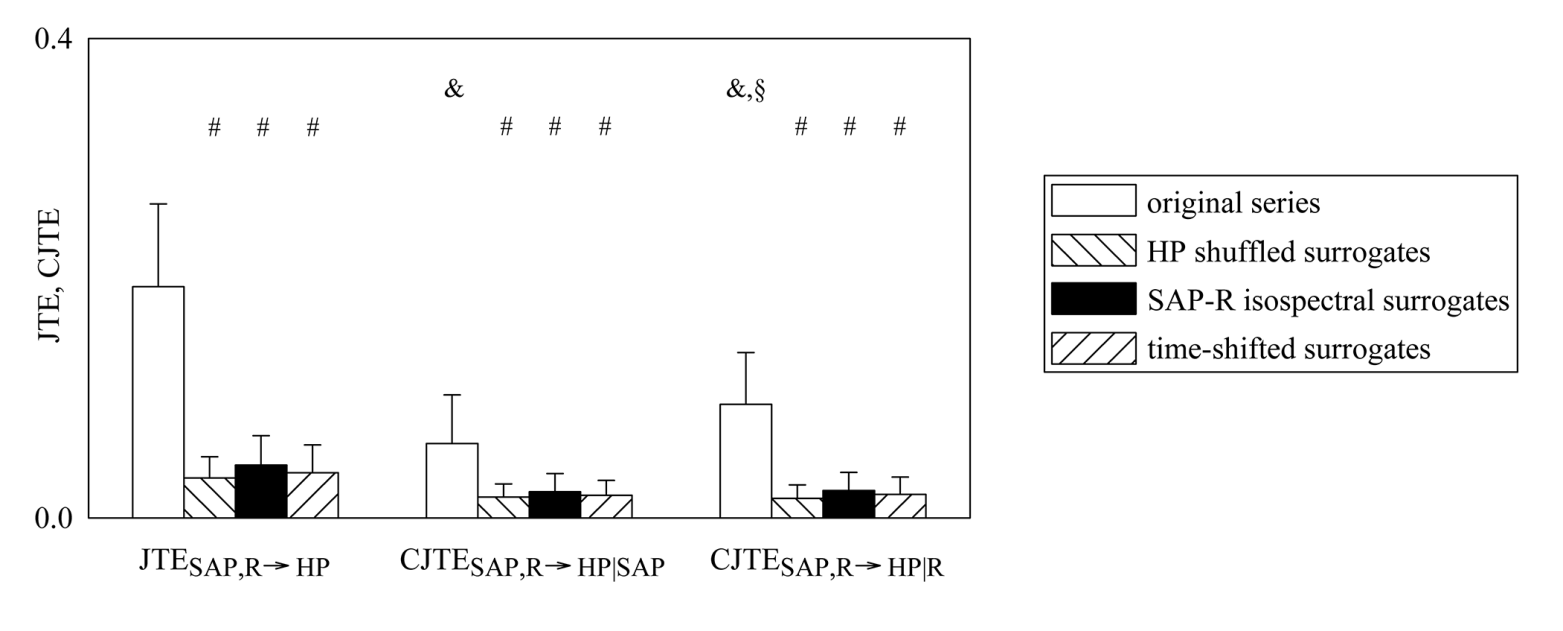
Comparison between JTE_SAP,R→HP_, CJTE_SAP,R→HP|SAP_ and CJTE_SAP,R→HP|R_. Grouped bar graph compares JTE_**SAP,R→HP**_, CJTE_**SAP,R→HP|SAP**_ and CJTE_**SAP,R→HP|R**_ computed during graded head-up tilt protocol over the original data (white bars), HP shuffled surrogates (backslash pattern bars), SAP-R isospectral surrogates (back bars) and time-shifted surrogates (slash bars). The values are pooled together independently of the tilt table inclination and reported as mean plus standard deviation. Within the same index (i.e. JTE_**SAP,R→HP**_, CJTE_**SAP,R→HP|SAP**_ or CJTE_**SAP,R→HP|R**_) the symbol # indicates a significant difference between original and surrogate series. Within the original data the symbols & and § indicate a significant difference versus JTE_**SAP,R→HP**_ and CJTE_**SAP,R→HP|SAP**_ respectively.

The grouped bar graph shown in Fig 6 compares JTE_SAP,R→HP_, CJTE_SAP,R→HP|SAP_ and CJTE_SAP,R→HP|R_ computed during head-up tilt protocol over the original data (white bars), HP shuffled surrogates (backslash pattern bars), SAP-R isospectral surrogates (black bars) and time-shifted surrogates (slash pattern bars). The values were pooled together independently of tilt table inclination. JTE_SAP,R→HP_, CJTE_SAP,R→HP|SAP_ and CJTE_SAP,R→HP|R_ were significantly different from 0 in the original data, while they were negligible in the case of all types of surrogates. Within the original data we found that JTE_SAP,R→HP_ was larger than CJTE_SAP,R→HP|SAP_ and CJTE_SAP,R→HP|R_, and CJTE_SAP,R→HP|R_ was higher than CJTE_SAP,R→HP|SAP_. Within the same index (i.e. JTE_SAP,R→HP_, CJTE_SAP,R→HP|SAP_ or CJTE_SAP,R→HP|R_) the markers computed over the original data were significantly higher than the same quantity calculated over surrogates.

**Fig 7:**
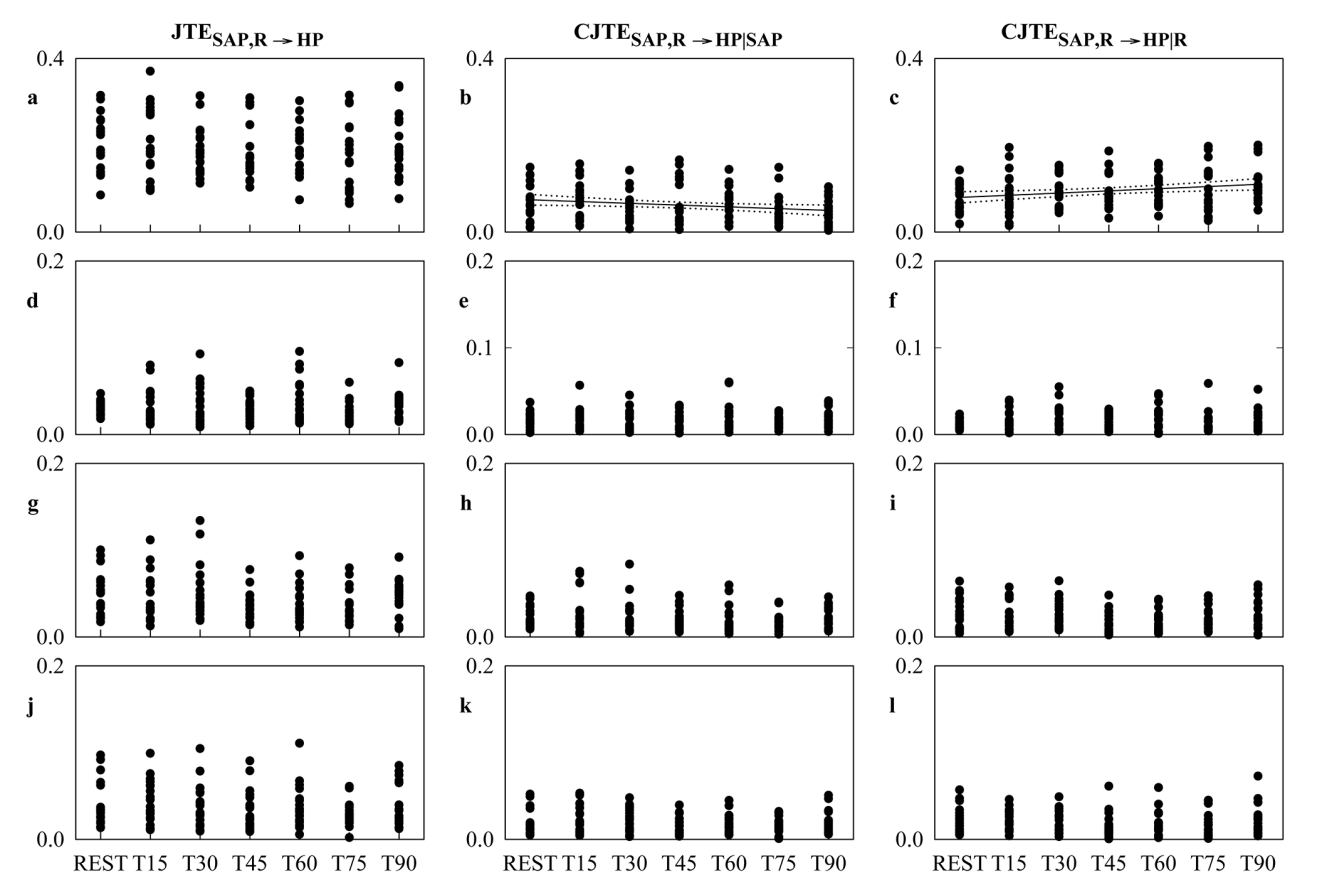
JTE_SAP,R→HP_, CJTE_SAP,R→HP|SAP_ and CJTE_SAP,R→HP|R_ during Graded Head-up Tilt. Individual values (solid circles) of JTE_**SAP,R→HP**_, CJTE_**SAP,R→HP|SAP**_ and CJTE_**SAP,R→HP|R**_ as a function of the tilt table inclination computed over the original data (a-c), HP shuffled surrogates (d-f), SAP-R isospectral surrogates (g-i) and time-shifted surrogates (j-l). When the slope of the regression line is significantly larger then 0, the linear regression (solid line) and its 95 percent confidence interval (dotted lines) are plotted as well. A significant positive correlation on tilt table angles is found only over the original data in the case of CJTE_**SAP,R→HP|SAP**_ (b) and CJTE_**SAP,R→HP|R**_ (c).

Fig 7 shows the individual values (solid circles) of JTE_SAP,R→HP_, CJTE_SAP,R→HP|SAP_ and CJTE_SAP,R→HP|R_ as a function of the tilt table inclination in the original data (Fig 7A–7C), HP shuffled surrogates (Fig 7D–7F), SAP-R isospectral surrogates (Fig 7G–7I) and time-shifted surrogates (Fig 7J–7L). The linear regression (solid line) and its 95 percent confidence interval (dotted lines) are plotted when the slope of the regression line is significantly larger then 0. When the linear regression analysis was performed over the original data, JTE_SAP,R→HP_ was unrelated to tilt table inclination (Fig 7A). Conversely, CJTE_SAP,R→HP|SAP_ (Fig 7B) and CJTE_SAP,R→HP|R_ (Fig 7C) were significantly related to tilt table angles (p = 1.99^.^10^−2^ and p = 6.71^.^10^−3^ respectively). The correlation coefficient was negative in the case of CJTE_SAP,R→HP|SAP_ (r = -0.202) and positive in the case of CJTE_SAP,R→HP|R_ (r = 0.234). When the linear regression analysis was performed over surrogates, JTE_SAP,R→HP_, CJTE_SAP,R→HP|SAP_ and CJTE_SAP,R→HP|R_ were unrelated to tilt table angles regardless of the type of surrogates (Fig 7D–7L).

## Discussion

The study computes PE, SE, CSE, JTE and CJTE over HP dynamics when SAP and R are considered as exogenous inputs during a protocol evoking a gradual sympathetic activation and vagal withdrawal.

From a methodological standpoint the main findings of the study can be summarized as follows: a) CSE, obtained by conditioning SE on all possible driving signals, is a measure of information internal to the target more specific than the information storage quantified by SE because it accounts exclusively for information intrinsic to the effect signal and excludes the contribution of the exogenous signals inflating the information storage; b) CJTE is a measure of information transfer more specific than JTE because it cancels joint direct influences on the target signal arising from driving signals utilized to condition JTE; c) CJTE is a measure of information transfer more flexible than complete (or partial) TE because it allows the assessment of the information jointly transferred from a subset of sources, selected among the whole set of sources according to some criteria, to the destination.

From an experimental standpoint the findings of this study can be summarized as follows: i) the PE of HP grows gradually with tilt table inclination; ii) the PE of HP is eliminated by destroying both the HP autocorrelation and coupling of HP to SAP and R and it is reduced by destroying the coupling of HP to SAP and R but preserving the HP autocorrelation; iii) the SE of HP is significantly larger than the CSE of HP given SAP and R; iv) the SE of HP increases progressively with tilt table angle and this dependence remains when CSE of HP given SAP and R is computed; v) destroying both the HP autocorrelation and coupling of HP to SAP and R eliminates SE of HP and CSE of HP given SAP and R and their dependence on tilt table angles; vi) destroying the coupling of HP to SAP and R but preserving the HP autocorrelation leaves unmodified the SE of HP and CSE of HP given SAP and R and preserves their dependence on tilt table angles; vii) the JTE from SAP and R to HP is significantly larger than CJTE from SAP and R to HP given SAP and CJTE from SAP and R to HP given R; viii) the JTE from SAP and R to HP is unrelated to tilt table angle; ix) the CJTE from SAP and R to HP given SAP gradually decreases with the magnitude of the orthostatic challenge, while CJTE from SAP and R to HP given R progressively increases; x) destroying the coupling of HP to SAP and R reduces to 0 both JTE and CJTE and eliminates the dependence of CJTE on tilt table inclination.

### SE and JTE are Unspecific Measures of Internal and Transferred Information

The present study confirms over experimental data that SE, i.e. a popular measure of information storage [3], depends in large part on the internal dynamics of the assigned output signal. Indeed, after destroying cross-correlation, wiping out autocorrelation eliminates SE, while preserving it leaves a significant amount of SE. Unfortunately, SE can be inflated by the contributions due to the action of the presumed sources as well [3,6], thus making it a quite unspecific measure of the information internal to the target. This dependence of SE on the presumed causes limits its interpretation because the effects of the sources cannot be dismissed. For example, in the specific context of this study the progressive raise of SE of HP with tilt table angles might be fully explained by the gradual raise of the contributions of SAP and R to the HP internal dynamics. This observation prompts us to render SE more specific by conditioning it on all the presumed causes via the CSE, thus eliminating direct influences of all sources from the information storage and obtaining a specific measure of the internal information solely related to the target dynamics.

In addition, the present study proves over experimental data that JTE might be insufficient for describing the information transferred to the destination. Indeed, the assessment of the information transfer from joint influences to the target might obscure that occurring from a specific source to the destination. For example, in the specific context of this study the JTE from SAP and R to HP is independent of the magnitude of the stimulus and hides the increase of information transferred to HP solely due to SAP, quantified by the CJTE from SAP and R to HP given R, and the decrease of information transferred to HP solely due to R, quantified by CJTE from SAP and R to HP given SAP. Therefore, JTE might necessitate to be conditioned on a specific subset of the sources to become a helpful measure of information transfer. This operation does not necessarily mean to compute the so-called complete (or partial) TE [1,11,28] that implies conditioning on the entire set of sources but the one setting the origin of the information transfer. Conversely, it means to condition on a specific subset of causes selected among all possible ones to cancel confounding factors capable to produce effects that are not primary driven by the mechanism under scrutiny, thus increasing the flexibility of complete (or partial) TE via the CJTE.

The differentiation between TE, JTE and CJTE might appear at the first sight a little bit labored. Indeed, the simpler notation TE_*x*→*y*|*z*_, where *y* is scalar, *x* and *z* can be multivariate variables with *z* that can be even null, can subsume all the above mentioned sub-genres of TE. However, the exploitation of the compact notation TE_*x*→*y*|*z*_ might obscure differences among information-theoretic quantities that the adopted terminology should help to put in evidence. These differences and the diverse specificity of indexes are outlined by the experimental results obtained during head-up tilt. This observation holds even in the case of SE and CSE.

### Physiological Correlates of the PE in HP Variability

PE represents the amount of uncertainty about the current value of the assigned target signal that can be resolved using past values of the same signal and past (and eventually present) values of all driving series in *Ω* (Fig 1C). Equivalently, PE represents the amount of information contained in the past values of the destination signal and past (and eventually present) values of all source signals in *Ω* about the current value of the destination signal. As expected, destroying HP autocorrelation and both HP-SAP and HP-R cross-correlation functions eliminates PE of HP and destroying both HP-SAP and HP-R cross-correlation functions but preserving HP autocorrelation reduces significantly PE of HP, thus confirming that all mechanisms inducing a memory of the current HP values over past HP, SAP and R values, such as dynamical properties of the sinus node [40,41], cardiac baroreflex [42,43] and cardiopulmonary pathway [44,45], contribute to control HP dynamics by reducing the uncertainty about its future values.

PE of HP increased as a function of tilt table angles. This result is not surprising given that the predictability of HP based on history of HP, SAP and R raises with the magnitude of the orthostatic challenge [46]. In the present study the metric is different compared to that exploited in [46] because the index was calculated in the field of information dynamics, but the approximation related to the assumption of Gaussianity makes the computed quantity significantly correlated with that exploited in [46]. PE of HP increased during graded head-up tilt due to the reduction of complexity of the cardiac control associated to the vagal withdrawal and sympathetic activation induced by the orthostatic challenge (i.e. fast variations of HP dynamics driven by vagal neural inputs were gradually limited with tilt table inclination, while slow HP changes associated to the sympathetic drive became progressively dominant) [15–17]. Remarkably the linear relation of PE of HP on tilt table angles was lost in HP shuffled surrogates but it was maintained in SAP-R isospectral and time-shifted ones. Since all surrogates imposed the HP-SAP and HP-R uncoupling but the HP shuffled ones destroyed the HP autocorrelation while SAP-R isospectral and time-shifted surrogates preserved it, we conclude that the linear relation of PE of HP on tilt table inclination does not depend substantially on the action of the exogenous sources, but rather depends mostly on the internal HP dynamics. This conclusion is also confirmed by the linear trends of SE of HP and CSE of HP given SAP and R, that were lost in HP shuffled surrogates and preserved in SAP-R isospectral and time-shifted surrogates, and by the linear trends of CJTE from SAP and R to HP given SAP and given R, that were lost in all types of surrogates. More specifically, the disruption of the link from SAP and R to HP due to cardiac baroreflex and cardiopulmonary pathway is not sufficient to prevent the increase of predictability of HP dynamics with the magnitude of the orthostatic challenge. Thus, mechanisms inducing a memory of HP over its own past values independent of SAP and R, such as dynamical properties of the sinus node or control reflexes involving more directly the sympathetic branch of the autonomic nervous system compared to more vagally-mediated reflexes like cardiac baroreflex and cardiopulmonary pathways [43,47], might play a relevant role in setting the overall level of HP predictability and its dependence on tilt table inclination.

### Physiological Correlates of the SE and CSE in HP Variability

SE represents the amount of uncertainty about the current value of the assigned target signal that can be resolved using past values of the same signal (Fig 1D). In other words, SE represents the amount of information carried by past values of the destination signal about the current value of the same signal. It has been proposed as a measure of the amount of information stored in the destination signal and usually computed as the mutual information between the current value of the given target signal and its own past [3,6]. It depends on both internal dynamical properties of the destination due to some memory processing that has nothing to do with the driving signals and the action of the driving signals that can favor information storage inside the target signal [3]. Our analysis based on surrogates supports the dependence of SE of the target signal on dynamics totally internal to the destination and on actions of sources: indeed, SE of HP was eliminated in HP shuffled surrogates and it was significantly reduced in SAP-R isospectral and time-shifted surrogates.

We found that SE of HP grows as a function of the magnitude of the orthostatic challenge, thus suggesting that sympathetic activation and vagal withdrawal facilitates progressively the information storage in HP variability. The increase of SE of HP with tilt table inclination is not surprising. Indeed, it is just another way to detect the augment of the predictability of HP dynamics during head-up tilt observed using several linear and nonlinear metrics including conditional entropy and predictability indexes applied exclusively to HP dynamics [46,48,49]. Again the progressive increase of SE of HP results from the reduced contribution of fast temporal scales to the complexity of HP dynamics as a consequence of the gradual vagal withdrawal with tilt table inclination [15–17].

The present study explores another interesting information-theoretic quantity, namely the SE of HP conditioned on all the presumed causes present in *Ω* (in our case the SAP and R series). CSE of HP given SAP and R measures the information shared by the current value of the HP series and its own past that cannot be explained by SAP and R (Fig 1E). The SE of HP conditioned on SAP and R was significantly smaller than the SE of HP and it was not significantly different from the same quantity computed over SAP-R isospectral and time-shifted surrogates. These observations confirm over experimental data that the SE of HP dynamics includes contributions attributable to SAP and R. However, even though diminished compared to SE of HP, CSE of HP assigned SAP and R remained significantly different from 0, thus suggesting that HP dynamics cannot be completely explained as a result of the actions of SAP and R over HP, and that mechanisms imposing a dependence of the current HP value on the HP history unrelated to SAP and R play a significant role in cardiac regulation. More importantly, CSE of HP assigned SAP and R remained under autonomic control because it gradually increased with the magnitude of the orthostatic challenge. Since after conditioning on SAP and R dynamics direct influences of cardiac baroreflex and cardiopulmonary pathway over HP variability should be eliminated, the increase of the CSE of HP assigned SAP and R with tilt table angles might be owing to direct sympathetic influences on the sinus node, unmediated by the activation of baroreceptors and/or low pressure receptors, and/or to modifications of the sinus mode dynamical properties possibly linked to the sympathetic overactivation. Therefore, we suggest that CSE of HP dynamics given SAP and R might provide a marker of the sympathetic drive directed to the sinus node more specific than quantities derived from univariate analysis of HP variability [50] and, hopefully, less controversial [51–53]. We advocate a direct measure of sympathetic discharge to finally prove the previously reported conjecture: indeed, we expect that, if the CSE of HP given SAP and R was a valuable marker of the sympathetic drive, the CSE of HP dynamics conditioned on sympathetic activity in addition to SAP and R series would remain stable with tilt table angles.

### Physiological Correlates of JTE and CJTE in HP Variability

The JTE from SAP and R to HP represents the amount of uncertainty associated to the current value of HP series that can be resolved using jointly present and past values of SAP and R above and beyond that can be explained exclusively using past samples of HP (Fig 1F). In other words it is the amount of information carried by present and past values of SAP and R that is helpful to predict the current value of HP and cannot be derived from past values of HP. As expected, JTE from SAP and R to HP was abolished by destroying HP-SAP and HP-R cross-correlation functions (i.e. in all surrogate sets). We found that this quantity is unrelated to tilt table angles. However, this quantify is quite unspecific because it mixes the information transferred jointly from SAP and R to HP. In order to render it more specific we need to compute two quantities by conditioning the JTE on SAP and R respectively. The CJTE from SAP and R to HP conditioned on SAP assessed the contribution to JTE likely due to cardiopulmonary pathway because the information provided by SAP, indicative of the cardiac baroreflex control, was conditioned out (Fig 1G). We found that this information-theoretic quantity progressively decreased with tilt table inclination, thus suggesting that, after canceling the influences of cardiac baroreflex, information transferred from R to HP progressively decreased with the magnitude of the stimulus, probably in relation to a weakened cardiopulmonary coupling due to the gradual vagal withdrawal [47]. The CJTE from SAP to R to HP conditioned on R assessed the contribution to JTE due to cardiac baroreflex when the information provided by R, indicative of the regulation imposed by the cardiopulmonary pathway, was conditioned out (Fig 1H). We found that this information-theoretic quantity progressively increased with tilt table inclination, thus suggesting that, after canceling the influences of cardiopulmonary pathway, information transferred from SAP to HP progressively increased with the magnitude of the stimulus probably in relation to a gradually augmented involvement of cardiac baroreflex [14,47]. The two opposite trends of the CJTE from SAP and R to HP conditioned on R and that conditioned on SAP explained the lack of any tendency of the JTE from SAP and R to HP with tilt table inclination. As expected, the disruption of the HP-SAP and HP-R cross-correlation functions in all sets of surrogates led to the abolishment of both CJTE from SAP and R to HP conditioned on SAP and by R and to the loss of their dependence on tilt table inclination. It is worth noting that, even though the CJTE from SAP and R to HP conditioned on R and that conditioned on SAP are coincident in this application with the complete (or partial) TE from R to HP and from SAP to HP in *Ω* [1,11,28], the CJTE is a quantity more flexible than complete TE because it allows the exploration of the information transfer from a more general subset of sources compared to the one assumed to be responsible for the complete (or partial) TE.

## Conclusions

This study shows over real data that CSE is a measure of the information internal to the target more specific than SE and CJTE is a measure of information transfer more specific than JTE and more flexible than TE. Indeed, when applied to HP dynamics derived during a protocol imposing a graded sympathetic stimulus, the CSE was found to be more powerful than SE in excluding possible influences of regulatory mechanisms on the HP information storage (i.e. the effect of cardiac baroreflex and cardiopulmonary pathway) and CJTE was more helpful than JTE in dissecting out relevant physiological mechanisms (i.e. the influences of the cardiac baroreflex can be separated from those of the cardiopulmonary pathway and vice versa). We conclude that the adopted information-theoretic quantities appear to be very powerful to derive information about the cardiovascular control from spontaneous HP, SAP and R variations in healthy subjects and deserve applications over pathological populations and in clinical settings to check their capability in describing deviations from the normal behavior, monitoring effect of treatment, predicting outcome and differentiating pathological states. Since nonlinear dynamics in cardiovascular variability are likely, even though less important in healthy subjects in experimental conditions enhancing the sympathetic drive [54,55], we advocate studies comparing the linear approach proposed in the present study, with model-free, nonlinear, approaches [5,8,9] to check the actual contribution of nonlinear dynamics to short-term cardiovascular control.
